# Epitopes and Mimotopes Identification Using Phage Display for Vaccine Development against Infectious Pathogens

**DOI:** 10.3390/vaccines11071176

**Published:** 2023-06-29

**Authors:** Marco Palma

**Affiliations:** 1 Institute for Globally Distributed Open Research and Education (IGDORE), 03181 Torrevieja, Spain; marco.palma@igdore.org; 2 Protheragen Inc., Ronkonkoma, NY 11779, USA

**Keywords:** epitopes, mimotopes, phage display, vaccine, vaccine development, pathogens, phages, bacteriophages, infections, immunization

## Abstract

Traditional vaccines use inactivated or weakened forms of pathogens which could have side effects and inadequate immune responses. To overcome these challenges, phage display has emerged as a valuable tool for identifying specific epitopes that could be used in vaccines. This review emphasizes the direct connection between epitope identification and vaccine development, filling a crucial gap in the field. This technique allows vaccines to be engineered to effectively stimulate the immune system by presenting carefully selected epitopes. Phage display involves screening libraries of random peptides or gene/genome fragments using serum samples from infected, convalescent, or vaccinated individuals. This method has been used to identify epitopes from various pathogens including SARS-CoV-2, Mycobacterium tuberculosis, hepatitis viruses, H5N1, HIV-1, Human T-lymphotropic virus 1, *Plasmodium falciparum*, *Trypanosoma cruzi*, and *Dirofilaria repens*. Bacteriophages offer advantages such as being immunogenic carriers, low production costs, and customization options, making them a promising alternative to traditional vaccines. The purpose of this study has been to highlight an approach that encompasses the entire process from epitope identification to vaccine production using a single technique, without requiring additional manipulation. Unlike conventional methods, phage display demonstrates exceptional efficiency and speed, which could provide significant advantages in critical scenarios such as pandemics.

## 1. Introduction

Epitopes are crucial components in the development of effective vaccines against infectious pathogens. These regions on the antigen’s surface engage with antibodies, B-cell and T-cell receptors, and serve as the portions of a pathogen that the immune system identifies and attacks. By identifying and selecting the most potent epitopes, it becomes feasible to design vaccines that trigger a strong immune response against the pathogen, while minimizing harm to the host [1,2].

Phage display technology shows great promise as an approach for identifying and selecting epitopes. This technique enables the screening of extensive libraries of peptides or proteins against a specific target, such as a pathogen, facilitating the identification of highly specific epitopes. The successful utilization of phage display has been observed in the identification and selection of epitopes across a wide range of pathogens, encompassing viruses, bacteria, and parasites. Once these epitopes have been identified, they can be harnessed for the development of vaccines that precisely target the pathogen in question [3]. Notably, phage display has proven effective in identifying epitopes from the spike protein of the severe acute respiratory syndrome coronavirus 2 (SARS-CoV-2) [4], which have been used for the development of COVID-19 vaccines and diagnostic tests [5]. Furthermore, phage display has been utilized to identify epitopes derived from *Mycobacterium tuberculosis*. These epitopes not only hold value in the realm of Tuberculosis (TB) serodiagnosis but also play a crucial role in the development of a potential TB vaccine [6].

The objective of this study is to provide a comprehensive overview of epitope identification, covering various aspects such as the different techniques employed, their respective advantages and disadvantages, and the utilization of phages in the process. Additionally, it will delve into the procedure of epitope or mimotope selection using phage display, examining both the general applications for selecting pathogenic epitopes and specific applications within the realm of infectious diseases. Lastly, the study will explore the role of phages in the development of vaccines.

## 2. Materials and Methods

### 2.1. Search Strategy

A literature review was conducted to address various topics related to epitope identification using phage-display. The review involved searching for articles in PubMed (https://pubmed.ncbi.nlm.nih.gov/, accessed on 25 April 2023) using the keywords “phage display”, “techniques”, or “approach”, combined with “epitope identification” or “epitope mapping”, specifically in the title or abstract.

### 2.2. Study Selection, Inclusion, and Exclusion Criteria

Non-English studies were excluded from the review, as well as letters, conference abstracts, and editorials. The review focused on articles that provided clarification on relevant technologies, protocols, serum samples, and phage-based vaccines.

## 3. Results

### 3.1. Techniques for Epitope Identification

Epitope identification encompasses a range of techniques that can be utilized based on the specific application and the type of epitope or mimotope under investigation. In certain cases, a combination of techniques may prove beneficial to attain a more comprehensive understanding of the epitope or mimotope in question. Some of the techniques commonly employed for this purpose include:

#### 3.1.1. X-ray Crystallography and Nuclear Magnetic Resonance (NMR) Spectroscopy

These techniques involve determining the three-dimensional structure of the antigen-antibody complex and aid in identifying the specific regions of the antigen recognized by the antibody [7,8]. The advantages of these techniques lie in their capacity to identify conformational epitopes, ascertain precise binding interactions between antibodies and antigens, and provide high-resolution structural information. In addition, NMR can be used to identify conformational epitopes by analyzing the interactions between an antibody or serum sample and a protein or protein complex in solution. However, X-ray crystallography faces challenges regarding protein solubility, which makes protein crystallization difficult [9] and the need to produce high-quality protein or protein complex crystals. As for NMR, it requires the production of high-quality isotopically labeled proteins. With both techniques, there is a risk that the results may not reflect epitope recognition in vivo, be costly, and require specialized equipment and expertise.

#### 3.1.2. Cryo-Electron Microscopy (Cryo-EM)

Cryo-EM is a powerful technique used for epitope identification, particularly in the field of structural biology. It allows for the visualization of macromolecular complexes at high resolution, including the interaction between antibodies and their epitopes [10,11].

Cryo-EM enables epitope identification by preparing and flash-freezing an antibody-epitope complex, imaging it using an electron beam, and recording 2D projection images. Through computational image processing, these images are aligned into a 3D reconstruction, allowing for the localization of the epitope region within the complex by analyzing the density map and comparing it to the known antibody structure. Epitope identification using cryo-EM provides high-resolution structural information in native-like conditions, aiding in understanding antibody-epitope interactions. However, cryo-EM requires technical expertise and specialized equipment, and sample heterogeneity can pose challenges during image processing.

Cryo-EM offers advantages for epitope identification, including high resolution that enables precise localization of epitopes and reveals atomic details of antibody-epitope interactions. Cryo-EM allows imaging in near-native conditions, preserving the natural conformation of samples without size limitations, making it suitable for a wide range of complexes. However, cryo-EM requires technical expertise and specialized equipment, making it costly and less accessible. Additionally, sample heterogeneity can pose challenges in achieving high-resolution reconstructions, potentially affecting epitope identification.

Despite these challenges, cryo-EM has revolutionized structural biology and has become an invaluable tool for epitope identification. Its ability to provide high-resolution structural information in near-native conditions makes it a promising technique for studying antibody-epitope interactions and facilitating the development of novel therapeutics.

#### 3.1.3. Peptide Arrays

Peptide arrays typically consist of a series of short peptides, each representing a potential epitope of the antigen of interest, and helps to recognize binding sites between proteins [12,13,14,15]. By incubating the peptide array with sera or cells from an individual with a known immune response to the antigen, it is possible to identify the specific peptides that are recognized by the immune system. Peptide arrays can be used in several ways to identify epitopes. One approach is peptide scanning that involves overlapping peptides that span the entire antigen sequence. This allows for the identification of multiple potential epitopes, as well as the determination of specific amino acid residues that are important for antibody recognition [16,17,18]. Another approach is to use a smaller set of peptides that are based on predicted or known epitopes. This technique offers several advantages. It enables simultaneous screening of a vast number of peptides and facilitates the detection of linear epitopes. However, there are certain limitations to consider. Firstly, it may not accurately reflect epitope recognition in vivo. Additionally, its sensitivity and specificity are relatively lower compared to full protein microarrays. Furthermore, the synthesis of numerous distinct sequences for analysis is not only time-consuming but also incurs substantial expenses [19].

#### 3.1.4. Site-Directed Mutagenesis

The technique involves targeting specific amino acids within the antigen sequence to alter them and determine their interaction with a specific ligand [20,21,22]. The first step in site-directed mutagenesis is to identify the candidate amino acids that are likely to be part of the epitope. This can be done by analyzing the structure of the antigen or by using peptide arrays or other experimental techniques. Once the candidate amino acids have been identified, they can be mutated using various methods, such as PCR-based mutagenesis or recombinant DNA technology. After producing the mutant antigen, it can be tested to determine its recognition by specific antibodies using techniques such as enzyme-linked immunosorbent assays (ELISA) or flow cytometry. If these antibodies no longer bind to the mutated antigen, it can be concluded that the mutated amino acid plays a crucial role in epitope recognition. On the other hand, if the antibodies still recognize the mutated antigen, it can be inferred that the mutated amino acid is not essential for epitope recognition. Site-directed mutagenesis can be used in conjunction with other techniques, such as peptide arrays or X-ray crystallography, to obtain a more complete understanding of the epitope. By identifying the specific amino acids that are critical for epitope recognition, site-directed mutagenesis can be used to design more effective vaccines or therapeutics, or to gain insights into the basic mechanisms of immune recognition.

This technique offers versatility as it can be applied to a wide range of antigens and can provide valuable insights into both the structural and functional aspects of the epitope, as well as the mechanisms of immune recognition. However, site-directed mutagenesis also has certain disadvantages. One drawback is that it can be a labor-intensive and time-consuming process, involving multiple rounds of mutagenesis, screening, and characterization. Moreover, the cost of site-directed mutagenesis can be high, particularly for larger or more complex antigens. Additionally, it is important to note that site-directed mutagenesis may not identify all the amino acids that are crucial for epitope recognition, as some amino acids may have functional redundancy or may be part of a larger epitope network.

#### 3.1.5. Bioinformatics and Computational Modeling

Bioinformatics, commonly referred as “in silico” in biology and other experimental sciences, can be employed to predict potential linear and conformational epitopes by analyzing the amino acid sequence and structure of a protein or protein complex [23,24,25]. Various algorithms and software tools are available for this purpose [26]. The technique can rapidly analyze large datasets of protein sequences, structures, and interactions to identify potential epitopes. It can provide highly accurate predictions of epitope binding sites based on protein structure and interactions and has the potential to be utilized for a diverse array of antigens, encompassing proteins, peptides, and even complete cells or viruses. In addition, it can be more cost-effective than traditional experimental methods, as they require fewer resources. Some of the disadvantages include limitations in the accuracy of computational models used for epitope identification due to the quality and completeness of the input data, as well as the assumptions and simplifications made in the model. Validation through experimental techniques, such as ELISA or flow cytometry, is necessary. False positives can arise, necessitating additional experimental validation and screening for confirmation. Specialized technical expertise and resources, including computational infrastructure and programming skills, are required.

#### 3.1.6. Phage Display

**Figure 1:**
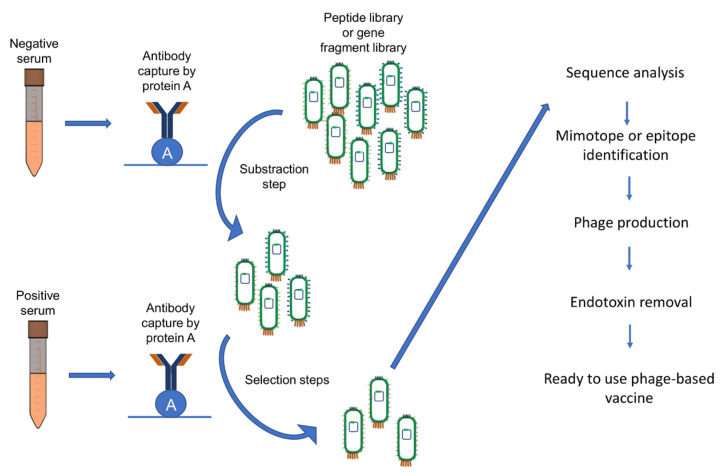
General strategy from epitope selection to phage-based vaccine production.

This technique is commonly used to identify and study protein-protein interactions and involves a library of modifying bacteriophages that display peptides or proteins on their surface, which can then be screened to identify epitopes recognized by a specific antibody. The phage library can be generated in different ways, such as through random peptide synthesis or PCR amplification of DNA fragments. During screening, the phage library is incubated with the antibody of interest and the particles that bind to the antibody are isolated and sequenced to identify the recognized peptides [27,28,29]. One advantage of phage display is that the selected phage clones can be used directly as a vaccine without additional manipulation, reducing the cost and time of vaccine development [30,31,32]. The general strategy from epitope selection to phage-based vaccine production is shown in Figure 1. Traditional approaches for identifying epitopes, which rely on methods such as antibody production or peptide synthesis, have limitations in detecting diverse and complex epitopes. In contrast, phage display offers multiple advantages in identifying epitopes that traditional methods may overlook. These advantages encompass library diversity, conformational diversity, unbiased selection revealing novel epitopes, and high-throughput screening [33]. While phage display has been used in vaccine development against specific viruses like HIV, it’s important to note that identifying conserved immunogenic epitopes across different virus strains does not guarantee a universal vaccine for all related diseases. Each virus or group of viruses has unique characteristics and variations in epitopes and immune responses. While phage display can help identify conserved epitopes, it doesn’t imply a single vaccine can protect against all related diseases. Developing vaccines for different diseases caused by related viruses requires tailored approaches considering specific characteristics, genetic variations, and immune responses. Additional studies are needed to assess the effectiveness and specificity of identified epitopes for each virus individually.

The length and linear/cyclic nature of displayed peptides in phage display have a significant impact on the selection outcome. Shorter peptides offer increased flexibility but may have lower affinities and more nonspecific interactions [34], while longer peptides provide more interaction potential but may face constraints and hindrances. Cyclic peptides exhibit enhanced stability and rigidity [35], leading to improved affinities and resistance to degradation, whereas linear peptides offer flexibility and conformational diversity. Optimal peptide length and conformation vary based on the target and experimental conditions. Researchers must carefully consider these factors during library design to enhance the chances of identifying peptides with desired binding properties in phage display selections.

While phage display is a powerful technique for identifying epitopes, there are some disadvantages to consider. One potential issue is the risk of false positives or false negatives due to the complexity of the screening process [36]. This can occur if the phage library is not sufficiently diverse or if the screening conditions are not optimized for the specific antibody or epitope of interest. Additionally, the size of the phage library and the number of potential epitopes to screen can be a limitation, as large libraries may require extensive sequencing and analysis, and the screening process may miss rare or low-affinity epitopes. Another concern is the potential for bias in the selection of epitopes, as the phage library may not fully represent the natural repertoire of potential epitopes. Finally, the use of phage display may not be suitable for identifying certain types of epitopes, such as those that require post-translational modifications or conformational epitopes that are dependent on the native folding of the protein.

### 3.2. The Phage Display Library and the Samples for Epitope or Mimotope Selection

#### 3.2.1. Types of Phage Display Libraries

Phage display libraries can be categorized into various types, depending on their origin and complexity, allowing for the presentation of peptides, proteins, or analogous molecules that mimic antigens. The prevailing varieties of phage display libraries employed in epitope mapping include random phage display peptide libraries (RPL) and gene or genome fragment libraries (GFL) [27]. These libraries possess distinct attributes and play a crucial role as valuable tools in unraveling epitopes.

RPLs are generated by introducing random nucleotide sequences into the phage display vector, offering versatility in identifying both linear and conformational epitopes. This includes the discovery of novel epitopes that may not naturally occur in proteins. The two main types of RPLs are linear peptide libraries and restricted peptide libraries. In linear peptide libraries, the amino acid sequences of the peptides can vary widely, without being restricted to specific motif or structure. This broad variability allows for screening of a wide range of potential epitopes. The primary advantage of this type of library lies in its ease of production and diversity, enabling the identification of a broad range of potential binders. However, a limitation is that the peptides may not always adopt a biologically relevant conformation, resulting in inconsistent binding affinity or specificity to the target. On the other hand, restricted peptide libraries are collections of peptides that are designed to mimic specific regions of a protein or to contain specific amino acid substitutions. Among the various types of restricted peptide libraries are fixed backbone, constrained peptide [37,38], scaffold libraries [39], and domain-specific libraries, each offering unique advantages and drawbacks. These libraries are useful for identifying epitopes that recognize specific structural motifs, domains, or subdomains, as well as facilitating screening for ligands that bind to specific receptors. However, it is important to note some disadvantages of RPLs. They can potentially generate false positives and may overlook crucial epitopes that are not adequately represented within the library.

GFLs are generated by cloning DNA fragments that are randomly generated from a genome or cDNA fragments into the phage display vector. They are useful for identifying naturally occurring epitopes and epitopes that are specific to a particular tissue or cell type. One advantage is that GFL libraries can be used to identify epitopes that are difficult to produce as synthetic peptides, such as discontinuous or complex epitopes. Additionally, GFL libraries can be used to identify epitopes that are not linear and require a folded protein structure to be recognized. The drawbacks include the fact that they require more effort and time to generate, and the library may not be representative of the full range of proteins expressed by the tissue or cell type. Additional drawbacks include the inability of GFL libraries to generate certain discontinuous epitopes, the lack of information on crucial binding residues, and the limitation of identifying epitopes on non-protein antigens [40].

Each of these types of phage display libraries has its advantages and disadvantages, and the choice of library type depends on the specific research question, the nature of the target protein, and the available resources.

#### 3.2.2. Library Construction

The initial step involves designing a phage display library, especially when a pre-made library is not readily available. This library is constructed using a genetically engineered bacteriophage, which presents a diverse array of peptides on its surface. To achieve this, a bacteriophage that infects commonly used laboratory bacteria, such as *Escherichia coli*, is carefully selected. M13 is a frequently employed bacteriophage in phage display experiments.

The next phase entails generating DNA fragments that encode the desired peptides through PCR amplification. These fragments are then cloned into the genome of the bacteriophage using molecular biology techniques. A commonly utilized method involves integrating the peptides into a gene that encodes a surface protein, such as pIII or pVIII. Once the cloning process is complete, the phage particles are produced by growing them within bacteria and subsequently harvested and purified. It is important to note that there are two approaches to phage display libraries: one employing a bacteriophage vector and the other utilizing a phagemid.

Bacteriophage vectors are modified versions of the full phage genome and contain an antibiotic selection gene and a recombinant hybrid gene that encodes the foreign peptide or antibody fused to the coat protein. Due to the limited number of coat protein subunits, it’s desirable to display the foreign peptide or antibody at a low valency to make detection and analysis easier. This is achieved by expressing both the wild-type and hybrid genes, leading to a lower level of display. While bacteriophage vectors are unsuitable for displaying antibodies and antibody fragments because of their size, they’ve been successfully used in peptide phage display applications. The main advantage of using bacteriophage vectors is that they don’t require helper phages for phage particle propagation since their genome already encodes all necessary replication genes.

Phagemids are a type of hybrid vector that combines plasmid and filamentous phage features. They possess a plasmid replication origin that enables stable maintenance and propagation of the vector in bacterial cells. Additionally, they contain a portion of the filamentous phage genome that allows for the presentation of peptides or proteins on the phage surface. Typically, phagemids do not encode much or any coat proteins by themselves and instead rely on a helper phage for the necessary structural and functional proteins during the phage life cycle. The helper phage provides capsid proteins for phage particle formation, as well as proteins involved in phage replication and assembly. When the phagemid DNA is packed into the phage particle along with the helper phage DNA, it results in phage particles that present the peptide or protein of interest on their surface. Compared to phage vectors, one advantage of using phagemids for phage display is their ability to accommodate larger and more complex peptide or protein libraries. Moreover, phagemids can be propagated as plasmids, which facilitates the cloning and manipulation of DNA fragments [41].

#### 3.2.3. Types of Samples

Serum samples obtained from patients recovering from an infectious disease or individuals immunized with live attenuated or inactivated virus vaccines contain antibodies that recognize epitopes associated with the pathogen or the disease. These antibodies can be used to identify antigenic epitopes that are important for the immune response. Analyzing the B- and T- cell responses in these individuals could assist in the development of future vaccines and therapeutics. Therefore, serum samples can be employed to screen phage display libraries, enabling the selection of epitopes suitable for inducing neutralizing antibodies [32,42,43]. By recognizing the epitopes involved in binding to target cells, these antibodies can effectively prevent the infection [44,45,46]. Furthermore, the selected epitopes can then be further characterized to determine their antigenic properties and potential use, not only in vaccines but also in diagnostic tools. This approach has proven successful in the development of vaccines against various viral pathogens, including HIV, hepatitis C virus, and influenza virus. However, it is important to carefully control the quality of the serum samples to ensure that they are specific and representative of the desired population. Additionally, using serum samples from vaccinated individuals may be limited by the fact that the immune response induced by vaccines may differ from the natural immune response induced during an infection.

The use of serum samples may have potential drawbacks that need to be considered. One concern is the possibility of antibodies in the serum identifying epitopes that are not specific to the targeted virus or disease, resulting in inaccuracies and false positive results. Additionally, there may not be enough antibodies in the serum samples to identify all the relevant epitopes, leading to missed targets and false negative results. Variability in antibody levels and specificity within serum samples can arise from factors such as age, vaccination history, and health status. Another challenge is the potential for cross-reactivity of antibodies in serum samples with epitopes from related viruses or diseases, which can create confusion in identification and potential misinterpretation of results. Therefore, it is crucial to exercise caution when utilizing serum samples and consider employing alternative methods alongside them to ensure accurate results.

#### 3.2.4. Antibody Purification

To prevent the selection of clones that bind to proteins other than the target antibodies, it is crucial to eliminate the additional proteins present in serum along with the antibodies. Therefore, purifying the antibodies from both the serum samples that contain the desired antibodies and those from negative sera is essential. Protein A/G affinity chromatography is a common method for purifying antibodies from serum samples [47]. Protein A and Protein G are two bacterial proteins that have a high affinity for the Fc region of many antibodies. A column containing Protein A or G is used to capture the antibodies, while other serum proteins pass through. The antibodies can then be eluted from the column using a low pH buffer or other methods. When the antigen is known and available, affinity purification of antibodies from a serum sample can be effectively carried out using antigen-based chromatography [48]. This method involves utilizing the specific binding interaction between the antibodies of interest and the immobilized antigen on a chromatography column.

### 3.3. The selection Procedure

Prior to commencing the selection or biopanning process, it is crucial to develop and refine two protocols. The first protocol pertains to the selection of a varied mixture of ligands from the library, using either serum or purified antibodies. The second protocol involves conducting a screening assay [49,50] to assess the binding ability of numerous individual clones within the ligand mixture, employing phage ELISA.

#### 3.3.1. Biopanning

The developed assay is utilized for screening the phage display library, facilitating the antibody-based identification of specific peptides from a vast pool of peptides within the library [51]. The following steps are crucial in the process of biopanning for epitope selection:

- To facilitate the selection process, it is vital to immobilize antibodies appropriately. Antibodies of interest must be purified to prevent interference from other proteins present in serum samples. Similarly, negative sera antibodies should be prepared for subsequent subtractive selection.
- To efficiently eliminate phage-specific antibodies from serum samples prior to their subsequent use, the samples can be incubated on a surface that has been coated with M13 helper phages. In the case of using a different type of bacteriophage, such as T7, for library creation, the protocol employs T7 phages lacking an insert instead of helper phages.
- To eliminate non-specific binders within the library and clones that bind to the constant region of antibodies, a subtractive selection step is essential. Thus, before proceeding with the selection process, the library is incubated with immobilized purified antibodies from negative serum.
- Subsequently, the phage solution is carefully transferred to the chosen solid support, such as immunotubes, microtiter plates, or magnetic beads, all of which are pre-coated with immobilized positive antibodies. For example, one approach involves capturing biotinylated antibodies using streptavidin-coated beads, while another option is immobilizing antibodies using protein A.
- To counteract any remaining non-specific binders within the library, negative antibodies can be added to the solution in the tube. The library is then incubated for a specific duration as required.
- The phage solution is discarded, and to ensure the removal of non-bound phages, multiple washes are conducted.
- To elute the binding phages, specific elution with purified positive antibodies or non-specific elution methods, such as pH adjustment or the use of a chaotropic agent, can be employed. When utilizing pH alteration, it is crucial to neutralize the eluted solution to prevent peptide denaturation or any negative impact on the phage particles.
- Following specific elution, the eluted phages are utilized to infect *E. coli* cells, facilitating the generation of a new phage stock. In instances where the library has been constructed using a phagemid, infection with a helper phage is required to enable the replication of the phage particles. If a T7 library is used, the eluted T7 phage is used to infect a suitable host strain of *E. coli* together with the appropriate antibiotic. The mixture is then incubated until lysis occurs. After lysis, the solution is centrifuged and subsequently transferred to a new tube.
- The selection steps are repeated in several rounds, with each round incrementally enhancing the stringency of the selection conditions. This can be accomplished by decreasing the amount of target molecules used for coating or by increasing the number of washes conducted.

#### 3.3.2. Identification of Potential Hits with Phage ELISA

Following the selection procedure, the identification of potential hits involves screening hundreds or thousands of individual clones using phage ELISA [52,53,54]. For example, for bacteriophage M13, single bacterial colonies containing the phagemid are selected from agar plates and inoculated into 96-well flat-bottom plates, ensuring one clone per well, in the presence of appropriate antibiotics. After an overnight incubation, the clones are transferred to fresh medium with antibiotics and agitated until reaching an approximate OD600 of 0.5. Subsequently, the clones are infected with a helper phage, such as M13K07, and incubated with agitation for an additional 16–18 h.

To select bacterial cells carrying the helper phage genome, such as M13K07, kanamycin is added. After centrifugation, the supernatants are subjected to analysis in an ELISA screening assay to detect antibody binding to the peptide phage. In the case of using another phage, such as T7, plaques formed in soft agar are used to infect a suitable host strain of *E. coli*, along with the corresponding antibiotic. The mixture is then incubated until lysis occurs. Following lysis, the solution is centrifuged, and the resulting supernatant can be utilized in phage ELISA.

For phage ELISA, antibodies are immobilized on the wells of microtiter plates, blocked, and the supernatants containing the phages are added in a suitable buffer. The mixture is incubated for 1 h. Next, the phage solutions are discarded, and non-bound phage particles are eliminated through three washes. The bound phages are subsequently detected using peroxidase conjugated anti-M13 antibodies or anti T7 antibodies, depending on the phage type in the library. Positive clones are then sequenced and analyzed for enriched sequences, as well as for sequences showing similarity to proteins in databases and overlapping sequences. To determine the ability of neutralizing epitopes to neutralize pathogens, their efficacy should be tested.

#### 3.3.3. Optimization of Hits

Once hits are identified, they may be optimized to improve their properties, such as increasing their immunogenicity and affinity or specificity recognition by positive antibodies. This may be accomplished using site-directed mutagenesis to modify specific amino acids in the peptide sequence [55]. A new library can be created based on the sequences of the selected peptides and new selection can be done to obtain epitopes recognized with better affinity and specificity by the positive antibodies.

#### 3.3.4. Characterization of Hits

Finally, the hits need to be fully characterized to determine the binding of antibodies to them and their ability to induce an immune response and how good that immune response is. This may involve further biochemical or biophysical characterization, as well as in vivo studies to assess their efficacy and safety.

### 3.4. General Applications of Phage Display to Select Epitopes of a Pathogen

Phage display is an immensely powerful technology used for the selection of epitopes specifically recognized by the immune system in response to infections caused by viruses, bacteria, or parasites. The identified epitopes have a wide range of applications, some of which are elucidated below.

#### 3.4.1. Diagnostic Test Development

The epitopes of a pathogen have significant potential in the development of diagnostic tests, such as ELISAs or lateral flow assays, to improve their accuracy and efficiency. ELISAs employ these epitopes as capture antigens immobilized on a solid surface, allowing for the detection and quantification of specific antibodies present in patient samples. On the other hand, lateral flow assays incorporate the epitopes conjugated to labels (e.g., gold nanoparticles) that enable visual detection of the target pathogen specific IgG and IgM antibodies in patient samples.

Moreover, the utilization of phage display technology allows for the identification of epitopes that are conserved among different strains of a pathogen. This aspect is particularly valuable for developing diagnostic tests capable of detecting a wide range of pathogen variants [56]. By incorporating epitopes of a pathogen into diagnostic assays, the accuracy and efficiency of pathogen detection and identification are significantly enhanced.

These diagnostic assays provide crucial information to healthcare professionals, empowering them to make timely decisions regarding patient management and treatment strategies. The incorporation of pathogenic epitopes into these tests ultimately contributes to improved patient care and outcomes.

#### 3.4.2. Understanding Pathogen-Host Interactions

Phage display is an invaluable tool for generating peptide libraries that can be systematically screened against different cell types. This approach enables the identification of binding peptides or proteins that exhibit a strong affinity for specific receptors on the surface of host cells [57]. By studying the specific interactions between epitopes of a pathogen and host cell receptors, researchers gain a deeper understanding of the mechanisms involved in pathogen entry, host cell invasion, and immune evasion.

Furthermore, phage display allows for the investigation of how these epitopes impact host cell signaling pathways, gene expression, and other cellular processes. This comprehensive analysis offers valuable insights into the intricate molecular mechanisms governing pathogen-host interactions. These insights, in turn, hold the potential to pave the way for the development of novel therapeutic approaches targeting these interactions.

#### 3.4.3. Developing Therapeutics

The epitopes of a pathogen, once identified, can be used in therapy in several ways:

- Monoclonal antibodies represent a potent strategy for targeting pathogenic epitopes with high specificity. These antibodies are specifically designed to bind to a particular epitope, allowing for diverse therapeutic applications. By engaging with the epitopes of a pathogen, monoclonal antibodies can neutralize the pathogen, impede its interaction with host cells, or initiate an immune response against it. Notably, this approach has demonstrated promising results in anticancer treatments [58], by targeting specific molecular markers on cancer cells, leading to enhanced therapeutic outcomes and improved patient survival rates. Their ability to specifically bind to epitopes of a pathogen, block key interactions, and stimulate immune responses represents a remarkable advancement in precision medicine, offering new avenues for the development of effective and tailored treatments [59].
- Epitopes can be utilized as valuable targets for small molecule inhibitors, which are specifically designed to bind to these epitopes and disrupt the function of the pathogen. By binding to the epitope, small molecules can exert their inhibitory effects by various means, such as inhibiting enzymatic activity or blocking receptor interactions [60], serving as a potent strategy to disrupt the pathogen’s lifecycle and limit its impact on the host.
- Gene therapy presents a promising avenue where the epitopes of a pathogen can be integrated. One approach involves the incorporation of genetic material encoding the epitope into host cells. By introducing this genetic material, the host cells gain the ability to produce the epitope themselves, consequently eliciting an immune response against the pathogen [61,62,63]. This strategy offers several advantages. Firstly, it allows for the sustained production of the pathogenic epitope within the host, ensuring a prolonged immune response. Secondly, by targeting the production of the epitope directly within the host cells, gene therapy circumvents the need for external administration of epitopes, making it a potentially more convenient and controlled approach.
- The recognition of antigenic epitopes holds the utmost significance in the advancement of vaccine development [64,65,66]. These epitopes serve as the building blocks for designing vaccines that activate the immune system to generate antibodies capable of recognizing and neutralizing specific pathogens. By precisely targeting these antigenic epitopes, vaccines can achieve heightened effectiveness and specificity, while reducing the potential side effects often associated with traditional vaccines that employ whole pathogens or inactivated forms of the pathogen.

Incorporating epitopes of a pathogen into vaccine design offers several advantages. Firstly, it enables a more focused and targeted immune response, concentrating on the critical components of the pathogen responsible for causing infection. This approach leads to a more efficient and robust immune response. Moreover, epitope-based vaccines can be customized to address specific strains or variants of the pathogen, providing adaptability and versatility in the face of evolving infectious agents.

### 3.5. Applications of Phage Display to Select Epitopes in Infectious Diseases

Phage display has proven to be a successful technique for selecting epitopes in various infectious diseases. This section provides practical examples of epitope identification using two key components, a phage display peptide or genome fragment library and samples from infected, convalescent, or vaccinated individuals. This has been demonstrated through studies on a range of pathogens, including SARS-CoV-2, *Mycobacterium tuberculosis*, hepatitis A-E, H5N1 influenza, HIV, and human T-lymphotropic virus 1, *Plasmodium falciparum*, *Trypanosoma cruzi*, and *Dirofilaria repens* (See Table 1).

#### 3.5.1. SARS-CoV-2

Through biopanning with antibodies from two COVID-19 patients, a total of 36 enriched peptides have been identified from a phage display peptide library. Among these peptides, four motifs exhibited consensus residues that corresponded to two potential B-cell epitopes found on viral proteins of the SARS-CoV-2 virus. They were further validated with competitive antibody binding and serological detection assays [67]. Recent studies have revealed that the C662–C671 epitope of SARS-CoV-2 plays a crucial role in triggering the production of antibodies against the S protein. These findings hold tremendous potential, as they have been successfully implemented in a groundbreaking prototype of an aerosol-delivered targeted phage-based vaccine [68].

Through a meticulous screening process, a phage display library containing gene fragments of SARS-CoV-2 was subjected to intense scrutiny against plasma samples from COVID-19 positive patients. This rigorous investigation yielded fruitful results, as it successfully isolated and identified specific peptide sequences that exhibited a strong affinity for SARS-CoV-2 antibodies. To gain deeper insights into the nature of these peptide sequences, a comprehensive deep sequencing analysis was performed on the recovered phage. The results of this analysis shed light on the distribution and characteristics of the epitopes present within the identified peptides. It was revealed that the majority of these epitopes were concentrated in the spike protein and nucleocapsid (N) regions of the SARS-CoV-2 genome [69].

Furthermore, a recent study conducted by Ballmann et al. (2022) involved the construction of a phage display library for the SARS-CoV-2 genome. The aim of this study was to identify immunogenic epitopes that are enriched in COVID-19 patients. Notably, they successfully identified an immunogenic polypeptide located within the fusion peptide (FP) region of the spike protein. This polypeptide demonstrated prominent recognition by sera from individuals affected by COVID-19 [4].

#### 3.5.2. *Mycobacterium tuberculosis*

To identify mimic epitopes of *M. tuberculosis*, the preliminary purified TB-positive serum was employed as a target to screen a phage display random 12-peptide library. This screening process yielded two prominent 12-mer peptides: CS1 (SVSVGMKPSPRP) and CS2 (TMGFTAPRFPHY). These peptides were enriched from the library using the purified mixed tuberculosis-positive serum. The binding affinity of the selected phages to the mixed tuberculosis-positive serum was confirmed through enzyme-linked immunosorbent assay (ELISA) and dot immunobinding assay. Notably, the results revealed a robust binding affinity between the positive phages and the serum from individuals with tuberculosis, but not to TB-negative control samples. BLAST analysis revealed some homology of their sequences with the sequences of acetaldehyde dehydrogenase, transposase, and the sequences of some unknown proteins. The authors highlight a drawback attributed to the limitations of the sample source, which solely consisted of TB-positive sera obtained from patients with active TB. However, to enhance the assessment of the practicality of the acquired mimotopes, they plan to conduct further analysis using additional samples collected from patients with non-active TB [6].

The identified epitopes possess significant value not only in the field of TB serodiagnosis but also assume a vital role in advancing the development of a potential TB vaccine. By characterizing these epitopes, researchers can gain insights into the specific regions of *M. tuberculosis* that trigger an immune response.

#### 3.5.3. Hepatitis Virus

Antibodies derived from blood samples of hepatitis A patients and a phage display peptide library were used to identify mimotopes of the viral capsid proteins (VP1 and VP3) of the hepatitis A virus (HAV) [70]. The library employed for this purpose, known as J404 PDPL, consisted of randomized linear peptides displayed on the pVIII capsid protein of bacteriophage M13 [71]. Magnetic microbeads were utilized for antibody purification, using mouse anti-human IgG and serum from a recovering hepatitis A patient. One of the identified phages was recognized by 92% of hepatitis A positive sera and inhibited the attachment of human anti-HAV antibodies to HAV. Following exposure to these mimotopes, BALB/c mice that had been immunized produced antibodies that neutralized HAV.

In a study conducted by Zhang et al. (2001), blood samples were obtained from a 28-year-old patient in the recovery phase of acute viral hepatitis B. These samples were used to screen an M13 random peptide library. The library consisted of linear 12 amino acid peptides fused with the phage gIII coat protein. Prior to incubating the serum with the library, it was preincubated with the M13 helper phage to suppress any phage-specific antibodies. Through a competitive experiment, the researchers identified and validated two mimotopes that corresponded to the pre-S2 region of the hepatitis B virus surface antigen (HBsAg). Furthermore, when BALB/c mice were vaccinated with synthetic mimotopes, the generated antibodies exhibited a high level of specificity in comparison to the HBsAg epitopes [72].

Similarly, in another study by Folgori et al. (1994), mimotopes of surface (HBsAg) antigens of the hepatitis B virus (HBV) were isolated from a library of nonapeptides fused with the phage pVIII. The isolation process consisted of attaching immunoglobulins from HBV-infected patients to polystyrene beads. These beads were subsequently preincubated with M13K07 phages before being incubated with the library. Mice that were immunized with the selected phagotopes displayed a strong and specific immune response against HbsAg [73].

The search for mimotopes of the human hepatitis C virus (HCV) core protein involved screening a random peptide library with serum obtained from a patient who tested positive for HCV. Through this screening process, three distinct phagotopes were identified. These phagotopes demonstrated recognition not only by an anti-core human monoclonal antibody but also by 45 different sera derived from individuals who were infected with HCV [74]. This highlights their potential as valuable tools for diagnostic and therapeutic applications. Further research and characterization of these mimotopes could contribute to the development of improved diagnostic assays and potential immunotherapies against HCV.

In a different study, a dodecapeptide phage display library was screened using positive serum for hepatitis E virus (HEV) that was attached to protein G magnetic microbeads. The identified mimotopes displayed a sensitivity ranging from 95.2% to 100% and a specificity ranging from 81.5% to 95.8% in various assays. Notably, despite lacking similarity to the core sequence of the HEV ORF2 capsid, these mimotopes successfully blocked the interaction between the HEV antigen and anti-HEV serum. Structural analysis revealed that these mimotopes belonged to the P region of the ORF2 capsid, which is located close to the conformational epitopes of anti-HEV neutralizing monoclonal antibodies [75].

#### 3.5.4. Influenza Virus

Antibodies from H5N1 convalescent sera were utilized to select specific H5N1 epitopes through screening H5N1 gene-fragmented phage display libraries (GFPDL). The GFPDL expressed polypeptides as pIII fusion proteins and were screened using antibodies from H5N1 convalescent sera. Notably, the study identified several epitopes recognized by the convalescent sera but not by the control sera, including epitopes from the neuraminidase catalytic site, PB1-F2, H5 HA[(-10)-223], and M2 ectodomain [76].

In another study, sera from individuals immunized with the H5N1 A/Indonesia/05/2005 (clade 2.1) VLP vaccine, which was produced in Sf9 insect cells, were used. The study aimed to identify epitopes of the influenza virus hemagglutinin (HA1/HA2) and neuraminidase (NA) using a GFPDL library. The purified antibodies derived from these sera exhibited superior recognition of the oligomeric form of HA1 compared to its monomeric form. Moreover, these antibodies demonstrated NA-inhibiting activity, attributed to their binding to a C-terminal epitope in close proximity to the sialic acid binding site [77].

#### 3.5.5. HIV

In a study conducted by Scala et al. (1999), mimotopes were isolated from phage display libraries utilizing sera obtained from HIV-infected patients. Among the selected mimotopes, there were ones that accurately mimicked HIV epitopes generated during infection, specifically regions of gp120 C2 and gp41. These mimotopes were not only recognized by sera from SHIV-infected monkeys but also stimulated the production of neutralizing antibodies when administered to immunized mice [78].

Similarly, mimotopes of HIV-1 were identified from M13 phage libraries by employing sera from individuals infected with HIV-1 subtype B. These particular mimotopes effectively mimicked the V3 crown epitope of HIV-1, resulting in the production of antibodies that bound to V3 peptides [79].

In another notable study conducted by Palacios-Rodríguez et al. (2007), CxxKxxC sequences were identified from a C7C phage display library using IgG obtained from HIV-1-infected patients. These sequences exhibited a resemblance to the immunodominant epitope CSGKLIC found in the envelope gp41 glycoprotein. When administered as a mixture in mice, these sequences induced higher titers of antibodies against the HIV-1 epitope [80].

#### 3.5.6. Human T-Lymphotropic Virus 1

Machado et al. (2022), conducted a study aimed at identifying mimotopes of Human T-lymphotropic virus 1 (HTLV-1) epitopes. To accomplish this, they utilized a pool of IgG from HTLV-1 patients to screen a phage display library. The primary objective was to discover specific peptide sequences that could mimic critical regions of HTLV-1 antigens. This approach provided them access to a diverse repertoire of potential mimotopes through a random 12-mer peptide library. Consequently, they were able to screen and select peptides that closely resembled the targeted epitopes, displaying a high degree of similarity. Notably, the chosen mimotopes closely resembled epitopes found in the envelope protein (gp46), Tax protein, and HTLV-1 protease, demonstrating promising characteristics in terms of their potential immunological relevance. These mimotopes could effectively serve as surrogate antigens, triggering immune responses similar to those elicited by the authentic HTLV-1 antigens [81].

#### 3.5.7. *Plasmodium falciparum*

To identify mimotopes of *Plasmodium falciparum*-infected erythrocyte surface antigens, a study employed a phage display library. Antibodies that were extracted from a pool of sera from malaria-infected individuals using *P. falciparum*-infected erythrocyte were used to screen the phage display library. After multiple rounds of panning several phage clones were selected. Among the 23 randomly chosen sequenced clones, 13 unique sequences were identified, and 3 of these sequences showed similarity to membrane proteins known to exist on *P. falciparum*-infected erythrocyte. Interestingly, most of the phage clones (7 out of 8) selected after the 4th panning exhibited specific binding to IgG from malaria-infected individuals. This specific binding was confirmed using sera from 24 malaria-infected individuals and 11 from uninfected individuals. Notably, one phage clone was most frequently detected after the 4th panning, demonstrating stronger binding to IgG in all malaria-infected individuals samples compared to any serum from uninfected individuals. Moreover, a rabbit antiserum raised against the peptide expressed by this clone specifically recognized the surface of *P. falciparum*-infected erythrocyte, leading to their hemolysis [82].

#### 3.5.8. *Trypanosoma cruzi*

Teixeira et al. (2021) developed an integrated platform for genome phage display of the eukaryotic parasite *Trypanosoma cruzi* enables the identification of thousands of antigens recognized by serum samples from patients with Chagas disease. Previous studies have shown a correlation between levels of anti-T. cruzi antibodies in patients and the persistence of the parasite, as well as eventual disease resolution. Certain *T. cruzi* antigens have been proposed as indicators of therapeutic efficacy. Therefore, the antibody response map generated through genome phage display holds significant potential in identifying and improving new biomarkers for Chagas disease. While four of the selected epitopes identified in this study demonstrated a noticeable correlation with the corresponding IgG pools, none of them proved to be specific markers for disease status. However, the study successfully validated at least five epitopes that are novel and not found in the existing database. The genomic phage platform presented in this study is a reproducible and effective tool for the rapid simultaneous identification of epitopes and antigens, not only in Chagas disease but also in emerging/reemerging acute pathogens on a global scale [83].

#### 3.5.9. *Dirofilaria repens*

Through the utilization of phage display technology and a 12-mer peptide library, Pękacz et al., (2022) successfully identified epitopes that exhibited a high reactivity with IgG from sera of dogs infected with *Dirofilaria repens*. This innovative approach facilitated the discovery of highly immunoreactive peptides that can serve as novel diagnostic markers for *D. repens* infections [84].

**Table 1:** Overview of studies using phage display for identification of epitopes from pathogens.

Pathogen	Antigen	References
SARS-CoV-2	Spike, N	[4,68,69]
*M. tuberculosis*	Acetaldehyde dehydrogenase, transposase, and unknown proteins	[6]
HAV	VP1, VP3	[70]
HBV	HBsAg	[72]
HCV	Core protein	[74]
HEV	ORF2 capsid,	[75]
H5N1	neuraminidase, PB1-F2, H5 HA, M2	[75]
HIV	gp120 C2, gp41, V3	[78,80]
HTLV-1	gp46	[51]
*Plasmodium falciparum*	DBL3χ, PfEMP-1, erythrocyte membrane protein	[82]
*Trypanosoma cruzi*	Novel	[83]
*Dirofilaria repens*	Novel	[84]

### 3.6. Use of Phagotomes in Vaccines

Vaccine development traditionally involves using inactivated or attenuated forms of pathogens or their components to stimulate an immune response. However, these approaches can have drawbacks, including side effects or inadequate immune responses. To overcome these challenges, researchers have turned to phage display technology, which enables the identification of specific epitopes that can selectively target the pathogen, reducing the risk of side effects. By selecting peptide sequences that bind to a particular target, phage display facilitates the design of vaccines that effectively elicit an immune response against the target.

In this innovative approach, phage particles displaying the chosen epitopes are administered to patients, prompting their immune systems to recognize the epitopes as foreign and mount an immune response against them. This response can prove effective in combating the target, whether it be a virus or cancer cell.

Phage-based vaccines offer numerous advantages over traditional vaccines. They have the capacity to elicit both humoral and cellular immune responses, which are crucial for comprehensive protection against pathogens. Additionally, these vaccines are relatively easy to produce and cost-effective [31], making them an attractive option for large-scale immunization programs.

However, there are still challenges that need to be addressed to optimize the selection and delivery of epitopes in phage-based vaccines, ensuring their maximum effectiveness in generating a protective immune response. It is crucial to continue exploring methods that enhance the stability and immunogenicity of selected epitopes, while also developing strategies for the efficient delivery and presentation of phage particles to target cells. By overcoming these challenges, phage display technology holds immense promise for the development of highly targeted and effective vaccines against a wide range of diseases, including infectious diseases and cancers.

## 4. Discussion

The identification and selection of epitopes play a crucial role in the development of vaccines, diagnostic tests, and therapeutic antibodies. Various techniques are available for this purpose, including X-ray crystallography, nuclear magnetic resonance (NMR) spectroscopy, peptide arrays, site-directed mutagenesis, bioinformatics and computational modeling, and phage display. The choice of technique depends on the specific application and the type of epitope or mimotope under investigation. Among these techniques, phage display stands out due to its simplicity in epitope selection and screening. Moreover, the resulting phagosomes can be used as vaccines without requiring additional manipulation.

To successfully select epitopes or mimotopes using phage display, several important steps must be carefully executed. Firstly, a phage display library is needed to facilitate the selection of target epitopes. These libraries can either be constructed in-house or obtained as pre-made libraries, such as those provided by New England Biolabs. There are two main types of libraries commonly used: random phage display peptide libraries (RPL) and gene or genome fragment libraries (GFL). Each type of library has its own advantages and disadvantages, and the choice depends on factors such as the specific research question, the nature of the target protein, and the available resources.

Obtaining the appropriate source of antibodies is another critical aspect when selecting epitopes for the library. These antibodies can be derived from patients who are recovering from an infectious disease or individuals who have been immunized with a vaccine. It’s important to note that these antibodies need to undergo purification using protein A/G affinity chromatography or antigen-based chromatography, if the antigen is available, to ensure that selection is not biased against other components present in the samples. However, it should be acknowledged that serum samples typically contain polyclonal antibodies, which necessitates a screening assay following the selection process.

Before commencing the selection or biopanning process, two protocols must be prepared and optimized. The first protocol involves biopanning the library, wherein a pool of phage clones recognized by the antibodies is selected. The second protocol is employed to identify the most promising hits or binders of interest within this pool, usually through a phage ELISA or an inhibition assay.

In addition to the steps outlined in the biopanning process, it is essential to highlight the importance of performing a subtractive selection using antibodies from negative samples. This critical step aims to eliminate non-specific binders in the library and clones that may bind to the constant part of the antibodies. To mitigate any potential interference during the panning process, it is recommended to add these antibodies to the phage library. One approach to achieving this is by incubating the serum sample on a surface coated with helper phages (e.g., for phagemid M13) or the original phage without an insert (e.g., for T7 phage) This effectively removes phage-specific antibodies before further utilization.

After obtaining the selected hits, it is important to optimize them if needed, possibly through techniques like site-directed mutagenesis. Subsequently, a thorough characterization of the hits is necessary to assess their antibody binding capability, their potential to elicit an immune response, and the effectiveness of that immune response.

The epitopes of a pathogen identified through phage display have diverse applications, including the development of diagnostic tests, understanding pathogen-host interactions, and the creation of therapeutics such as monoclonal antibodies, small molecule inhibitors, and vaccines.

Moreover, numerous studies have employed phage display techniques in the context of infectious diseases, using two key components: a phage display library and antibodies from samples taken from infected, convalescent, or vaccinated individuals. For instance, in the case of hepatitis A virus (HAV), mimotopes were identified using antibodies from hepatitis A patients. One of the identified phages exhibited the ability to hinder the attachment of anti-HAV antibodies, eliciting a specific immune response in mice. Similarly, phage display libraries were utilized to discover mimotopes of the hepatitis B virus (HBV) surface antigen (HBsAg), leading to the generation of highly specific antibodies in vaccinated mice. Furthermore, mimotopes of the human hepatitis C virus (HCV) core protein were identified using serum from an HCV-infected patient. Comparable studies were conducted for hepatitis E virus (HEV), H5N1 influenza, HIV, and Human T-lymphotropic virus 1 (HTLV-1), resulting in the identification of mimotopes and the induction of immune responses. These studies exemplify the immense potential of phage display libraries in identifying specific epitopes for vaccine development, designed to be safe and effective in preventing the associated diseases.

The development of vaccines has traditionally relied on the use of inactivated or attenuated forms of pathogens. However, these methods have their limitations, such as side effects and insufficient immune responses. To address these challenges, phage display has emerged as a valuable tool for identifying specific epitopes that can selectively target pathogens, mitigating the risk of side effects associated with using whole pathogens. By harnessing the power of phage display, vaccines can be engineered to effectively stimulate the immune system by presenting carefully chosen epitopes.

It is worth noting that phage-based vaccines are not currently available in the market. Nonetheless, research and development in this area have shown promising results. For instance, bacteriophage vaccines based on M13 have demonstrated safety in both humans [85] and animals [86]. This approach offers the advantage of reducing the need for additional adjuvants, which are commonly used in traditional vaccines to enhance immune responses. By leveraging the natural immunogenicity of phage particles, these vaccines have the potential to provide effective protection against pathogens.

Another significant advantage of phage-based vaccines is their relatively low production cost. This makes them particularly suitable for regions with limited resources that are frequently affected by infectious diseases. These regions often face challenges in accessing and affording conventional vaccines. By developing and producing their own phage-based vaccines, these regions can gain more control over their healthcare systems and contribute to improved disease prevention and public health outcomes.

Additionally, phage-based vaccines offer the flexibility of customization. Researchers can select and optimize specific epitopes from pathogenic organisms, tailoring the vaccine to target the most relevant components for an effective immune response. This precision in epitope selection can lead to vaccines that are highly specific and potent in combating infectious diseases.

However, it is important to note that further research and development are needed to address certain challenges associated with phage-based vaccines. These challenges include improving the stability of phage particles, enhancing immunogenicity, and ensuring efficient delivery to target cells. Researchers are actively exploring various strategies to optimize these aspects and maximize the potential of phage display technology in vaccine development.

## 5. Conclusions

This comprehensive review illuminates the unique perspective of phage display, establishing a direct connection between epitope identification and vaccine development to address a specific gap in the field. In Section 3.5, practical examples of epitope identification are presented using two key components, a phage display peptide or genome fragment library and samples from infected, convalescent, or vaccinated individuals. Successful identification of pathogen epitopes has been achieved by using antibodies from these patients. However, there is currently no example where phage display-based epitope identification has been followed by direct utilization of the identified and isolated phage clone as a vaccine. Only one clinical trial has been conducted using M13 vaccine for cancer patients [85]. The purpose of this study has been to highlight an approach that encompasses the entire process from epitope identification to vaccine production using a single technique, without requiring additional manipulation. Unlike conventional methods, phage display demonstrates exceptional efficiency and speed, proving particularly advantageous in critical scenarios such as pandemics. Notably, the timeline from selection to preclinical or clinical trials is significantly compressed, enabling the acquisition of a viable vaccine product within a matter of months instead of years. The review underscores the numerous advantages offered by phage display, encompassing expedited processes such as panning, characterization, sequencing, phage production, and endotoxin removal. By bridging this crucial knowledge gap, the review delivers invaluable insights into the remarkable potential of phage display for swift vaccine development. Undoubtedly, phage-based vaccines have the power to revolutionize the field of vaccinology, boasting immunogenic carrier capabilities, cost-effectiveness, and customization options that position them as a promising alternative to traditional vaccines. While further refinement and validation remain imperative, the advancements in phage display technology hold immense promise for the creation of precise and potent vaccines against infectious diseases.
